# There was not, they did not: May negation cause the negated ideas to be remembered as existing?

**DOI:** 10.1371/journal.pone.0176452

**Published:** 2017-04-27

**Authors:** Józef Maciuszek, Romuald Polczyk

**Affiliations:** 1 Institute of Applied Psychology, Jagiellonian University, Cracow, Poland; 2 Institute of Psychology, Jagiellonian University, Cracow, Poland; University of Leicester, UNITED KINGDOM

## Abstract

In this article we demonstrate that negation of ideas can have paradoxical effects, possibly leading the listener to believe that the negated ideas actually existed. In Experiment 1, participants listened to a description of a house, in which some objects were mentioned, some were negated, and some were not mentioned at all. When questioned about the existence of these objects a week later, the participants gave more false positives for items that were negated in the original material than for items that were not mentioned at all, an effect we call negation related false memories (NRFM). The NRFM effect was replicated again in Experiment 2 with a sample of five and six year-old children. Experiment 3 confirmed NRFM in the case of negated actions. The results are discussed in terms of retention hypothesis, as well as the theory that negation can activate a representation of an entity and behaviour. It is also indicated that future research is needed to ensure that it is indeed negation which caused false alarms, not merely mentioning an object.

## Introduction

‘Everything she’s saying about me is rubbish and slander’, an upset politician told the local newspaper, ‘I didn’t beat my wife, I didn’t cheat on her, and I don’t abuse alcohol’.

Negation is an important part of our everyday communication; indeed, it is an integral part of it [1]. Common sense and everyday psychology assume that negation should always lead to an expected effect, namely, that when we deny the existence of something, our interlocutor will assume that the negated entities do not exist. We expect something similar, when we express opposition, resistance, or refusal (I don’t want it), or the desire to control somebody’s behaviour, or to prohibit or forbid something (Don’t smoke; Don’t go there; Don’t fall down; Don’t worry; Don’t think about…). What’s more, when we state that something does not exist or that something has not been done, we expect our interlocutors to remember the denial.

It was demonstrated several decades ago that processing negation sentences is more difficult than processing affirmative sentences [2,3,4,5,6,7]. Numerous counter-productive effects of negation can be found in the literature: e.g. in the political context [8], in the context of prohibitive signs [9], in research on questions and negation as tools of insinuation [10], and in studies on mental control mechanisms [11]. However, it is also important to underline that in the last several years a large of amount research has shown that a delay in negation processing can be alleviated by pragmatics and different semantic contexts [12, 13,14,15,16,17,18]. The context in which the negation occurs may play a primary role in children’s (2–5 years) comprehension of negation [19].

In this study, we experimentally tested whether recalling negated objects and actions can have unintended, ironic effects; instead of remembering that entities did not exist, they may be recalled as in fact existing, thus resulting in higher rates of false memory alarms. Such counterproductive effects of negations have obvious practical consequences. One of the more dramatic ones may be connected with applied forensic psychology. Imagine that a witness denied the existence of a weapon, and another witness, after hearing it, erroneously testifies that a weapon was present. In the present study we are interested in the paradoxical influence of negation on memory processes, namely, whether telling participants that something does not exist can make them erroneously ‘remember’ the negated ideas as existing.

Negation not only can have counter-productive effects in the communication context and make information processing and reasoning more difficult, but it also impedes remembering—sentences with negation often prove to be more difficult to remember than affirmative ones [20,21,22].

There are two main paradigms in research on the influence of negation on memory. The first one (‘memory for negated sentences’) entails investigating memory related to negation in comparison to memory related to affirmation, both of which are presented to participants prior to the memory tasks, e.g. [23,24]. Thus, in this paradigm, negations are present in the original to-be-remembered material and the number of correctly and falsely remembered negations is the main dependent variable.

The other paradigm investigates the influence of negating the existence of certain entities or features by research participants on the memory for these objects in a subsequent memory test [25,26]. Within this paradigm negation is not included in the original material (e.g. a video), but instead it is a reaction of a research participant to a question regarding the existence of certain objects [25] or object features in the first test of memory [26]. In these experiments participants watched a short film of a virtual tour of an apartment. After viewing the film, they took the first memory test in which they were asked whether different objects were present in the apartment [25] or, whether some entities (e.g. a carpet) had certain attributes (e.g. yellow) [26]. In Fiedler et al.’s study [25] participants correctly negated the presence of objects that were absent, and in Mayo’s [26] study participants correctly negated an attribute of objects that were actually present. After some time, in a second memory test, the participants were again asked whether different objects were present in the original material. It turned out that the number of false alarms was higher in this second test when non-present objects were correctly identified by participants as having not been present in the first test, compared to when non-present objects were not mentioned by the participants in the first test [25]. For example, correctly negating the presence of “an umbrella” in the first memory test led participants to mistakenly indicate the presence of an umbrella during the subsequent memory test.

Next, Mayo et. al. [26] found that correctly negating a feature of an entity led to greater memory loss for that entity compared to when the feature was correctly affirmed. For example, correctly negating that the carpet in the bedroom was blue led to a greater memory loss of that carpet compared to when it was correctly affirmed that the carpet was blue. Thus, the second paradigm may be called ‘the effect of correct denying on memory’.

The main question of studies conducted in the ‘memory for negated sentences’ approach is whether memory for sentences containing negation is different from memory for affirmative sentences. These studies show that, compared to affirmative sentences, memory performance for negation is generally poor [20,23]. Additionally, various moderators of the influence of negation on memory have been investigated. Some studies have focused on differences in memory in relation to the types of words encompassed by the negation, for example, uni-polar and bi-polar descriptions [22] and dichotomous and continuous antonyms [27]. Other studies have examined whether remembering negation depends on the syntactic structure of the sentence (coordinate sentences and complex sentences) [21] and the concreteness and abstraction of the sentences [28]. Research attention also has been given to whether inferring negation through recollection is easier in the case of antecedent propositions versus consequent propositions [29]. Other studies have compared the memorization of negated and affirmative sentences in various languages, such as in English and French [30].

Our research differs from both of the two aforementioned paradigms. It differs from the ‘memory for negated sentences’ paradigm in that the aim of the present research was not to analyse the memory of negations, but rather to compare the number of falsely recalled entities negated in the original material compared with a condition in which the items were not mentioned at all. The present research is therefore similar to this paradigm in that the negation is included in the original material itself. As for the second paradigm, the ‘effect of correct denying on memory’, our research is different from it in that the negation is included in the original material, not in any way outside it. Our research is however similar to this paradigm in that it explores whether negation can cause false alarms.

In a way, research on the misinformation effect can be viewed from the perspective of the connections among negation, memory, and counterintuitive research. In this paradigm [31,32], participants are usually presented with some original material (e.g. a video clip), after which they are given some sort of post-event material (e.g. a written summary of the video) in which, in the experimental condition, some items are incongruent with the video. In the final memory test, all participants answer a series of questions about the original video, including the critical items. It is now well established that within this paradigm the participants from the experimental conditions are less correct about the critical items than those from the control condition [33,34].

In the present experiments, we investigated whether a statement included in the original material that negates the existence of an object or an action may increase false memories for this object or action. We call this effect ‘negation related false memories’ (NRFM). Technically, it consists of a higher number of false-positive alarms for negated items than for items that were not mentioned at all. The basic idea was to present the participants with a narrative, then, either immediately or after a week, have them answer a series of questions related to the narrative. The narrative included three types of communication: an affirmative statement (e.g. There was a carpet in the room), a negative statement (e.g. There was no carpet in the room), and no statement (no sentence in the narrative about carpets being or not being in the room). The first (affirmative) condition was included for control reasons, and the comparison between the second and third statements constituted the core elements for diagnosing NRFM.

This approach differs from the aforementioned examples of post-event information studies, in which the negation was presented somewhere outside the original material. In contrast to those experiments, in the present research, the negations are present in the original to-be-remembered material. This allows us to examine a rather simple question, namely, whether people who were told that something does not exist, tend to remember it as actually existing.

In contrast to studies conducted within the traditional paradigms, we wanted to study the memory of a meaningful narrative, rather than separate unrelated sentences, as done in the aforementioned research, [21,22]. We also wanted to concentrate on long-term memory (i.e. one week later), and to compare it to memory immediately after exposure to the narrative in question. What’s more, we wanted to directly compare false memory for negated ideas with false memory for ideas which were not mentioned at all. Thus, the main aim of the present research was to investigate whether negation can have surprising and counter-intuitive effects on long term memory.

Apart from merely testing the existence of NRFM, we wanted to investigate some of its possible moderators, the first of which was the typicality of a given item. Inspiration for investigating the effect of typicality came from schematic memory models [35] (the effect of a schema on recollection), and Wason’s model of the semantic function of negation [2] (it should be noted that the semantic function of negation discussed by him is outside the scope of this paper). In general, schemata influence memory [36]. A range of factors determine whether information that is consistent with a schema is remembered better than information that is not consistent with it [37]. In Wason’s view [2], negation performs a special semantic function; it is used primarily when the normal course of events is altered, such as when something inconsistent with the beliefs or expectations of interlocutors occurs. For instance, the sentence “The train was not late this morning” is more justified if the train is usually late. We were interested in whether the negation of the presence of expected (typical) objects would have different effects on memory compared to the negation of the presence of objects that were not expected (atypical objects).

Also, it is well known that ‘atypical’ facts, i.e. those inconsistent with certain schemata and scripts, tend to be better remembered than typical facts. This has been labelled the ‘typicality effect’ [36,38,39,40]. Therefore, it is possible that the typicality vs. nontypicality of negated facts could be related to NRFM. It is also known that people tend to fill ‘gaps’ in memory with elements congruent, rather than incongruent, with schemata and scripts, e.g. [41,42]. This is particularly true in situations where the source material does not make any mention of something, which is explicitly referenced in a subsequent memory test. In the case of unmentioned ideas, typical ideas should be falsely reported more often as schemata and scripts are frequently used to fill in the gaps [43,44]. However, denying atypical, improbable or implausible ideas makes them more typical and congruent with expectations, whereas denying typical ones renders them not typical. Hence, the typicality of an idea may influence NRFM. More specifically, in light of the typicality effect mentioned above, in the immediate condition we expected better memory for atypical objects that were mentioned, compared with typical ones that were mentioned. However, some research suggests that the typicality effect disappears after some time [45]: after a week, the impact of the script was strong enough to counterbalance that of typicality. It may be that both effects are present simultaneously [37]. We predict therefore that there will be no difference between typical and untypical items as regards the number of correct answers. The same may be true in the case of the negations, thus we do not expect differences in the number of false alarms for negated typical and untypical items. In contrast, we expected more false alarms in the case of typical objects that were not mentioned.

Another variable that was manipulated in the experiments was the time delay between the presentation of the original narrative and the memory probe. For obvious reasons, a time delay makes remembering more difficult. We hypothesized that the NRFM effect would be present when there was a long time delay (one week), but not when the memory test was performed immediately after presenting the original narrative. We anticipated this pattern of results on the assumption that during the initial memory probe, memory for the negated particles would still be strong, thereby not yet allowing room for the effects of NRFM. It may be so because of the dissociation errors, e.g. Mayo [22] stated that ‘the core supposition could be dissociated from the negation tag at a later time’ (p. 434). So losing of the negation marker is less likely in the immediate condition.

### Overview of the experiments

In Experiment 1, the existence of NRFM was tested by presenting participants with an audio description of a newly-built house in which some objects were mentioned, some were negated, and others were not mentioned at all. Some of the objects were typical for a modern building and others were atypical. A memory test was performed either immediately following the presentation of the description or one week later. In Experiment 2, children were tested using a time delay of one day in order to replicate the findings of Study 1 on a different sample. Experiment 3 had the same basic structure, but instead of being about objects, the text was about the actions of a person driving a car.

All the materials were presented in the Polish language. The negating particle in Polish is ‘nie’. Efforts were taken to have sizeable sample sizes so as to provide adequate statistical power. As with increasing sample size even trivial effects may prove significant, we therefore also calculated effect sizes: Cohen’s partial eta-squares [46].

## Experiment 1

### Methods

#### Participants

Two-hundred and ninety-two participants, who were mainly first-year psychology students, took part in Study 1; 223 women and 60 men (nine participants did not indicate their gender). Their mean age was 20.5 (*SD* = 1.8). All participants volunteered to take part in the study, and they did not receive compensation (financial or otherwise) for their participation. The participants provided verbal informed consent to participate in the study. Written consent was not obtained in order to assure the declared anonymity of the participation. The procedure, including giving verbal consent, was approved by the Ethical Committee of the Institute of Psychology, Jagiellonian University.

#### Materials

1. An audio recording of 170 words, and with a duration of 1 minute and 8 seconds, described a house that was almost ready to live in. The text included some items that were explicitly mentioned or explicitly negated, e.g. ‘There is a living room in the house, but there is no porch’. There were two versions of the description; one contained items that are fairly typical for a house (such as a table, lamps, mirrors, and a windowsill), while the other contained items that are rather atypical for an average house (such as a sauna, a crane, and barbed wire). In each version of the description, six items were mentioned, six items were negated, and another six items were not mentioned at all, serving as controls in the memory test. Full counterbalancing was used so that each of the items appeared in each of the three conditions: mentioned, negated, and not mentioned. In sum, to assure full counterbalancing, there were six versions of each of the two conditions (typical and atypical items).

2. A memory test was conducted that consisted of 18 items; six of them were mentioned in the narrative, six were negated, and six were not mentioned. Depending on the counterbalanced version, a given item might have served for a given participant as a mentioned, negated, or control item (not mentioned). Each item was scored by the participants as ‘Present’ (that is, mentioned in the narrative as being present in the house) or ‘Not present’ in the house. There were two versions of the memory test: one for the narratives with typical items and another one for the narratives containing atypical items.

#### Procedure

Participants were tested in groups of 10 to 18 individuals. At the beginning of the experiment, they listened to descriptions of the house that were presented through earphones. In the immediate memory probe condition, they were given a short task that was intended as a filler to mitigate the memory ‘recency effect’ [47]. This task consisted of writing down the names of the days of the week and the months of the year in alphabetical order. Afterwards, participants were given the memory test, starting with the following instructions: ‘You have just listened to a description of a house. On the basis of that description, place an ‘X’ in one of the columns for each item–‘Present’ or ‘Not present’. In the delayed memory probe condition, the participants were dismissed after listening to the narrative, and they were given the memory test one week later.

#### Experimental design

There were two between-subject factors in the experiment: delay (immediate vs. one week) and the version of the narrative (typical vs. atypical items). NRFM was manipulated as a within-subject factor, with each of the participants listening and responding to the mentioned, negated items and not-mentioned ones. Thus, the design was as follows: delay (2: immediate vs. one week) × typicality (2: typical vs. untypical) × type of information (3: mentioned vs. negated vs. not mentioned). In Study 1, as in the subsequent studies, the number of ‘Present’ answers in the final memory test was the dependent variable. Thus, in the case of mentioned items, the dependent variable referred to correct answers; for negated items, ‘Present’ answers are false alarms; and for items that were not mentioned, ‘Present’ answers are also false alarms. Analysis of variance (ANOVA) was used to analyse the data.

### Results

The descriptive results across all experimental conditions are presented in Table 1.

**Table 1:** Means and standard deviations for the ‘Present’ answers across experimental conditions—Experiment 1.

		Means			Standard deviations			N
Delay	Typicality	Men	Neg	Con	Men	Neg	Con	
Immediate	Typical	4.95	1.58	2.26	1.10	1.34	1.40	78
	Untypical	5.21	1.74	1.39	0.90	1.47	1.71	80
	Total	5.08	1.66	1.82	1.01	1.41	1.62	158
One Week	Typical	3.54	3.15	2.56	1.12	1.41	0.97	39
	Untypical	3.62	2.88	1.17	0.94	1.19	1.12	42
	Total	3.58	3.01	1.84	1.02	1.30	1.26	81
Total	Typical	4.48	2.10	2.36	1.29	1.55	1.28	117
	Untypical	4.66	2.13	1.31	1.18	1.48	1.53	122
	Total	4.57	2.12	1.82	1.24	1.51	1.50	239

Note: Men = mentioned; Neg = negated; Con = control (not mentioned)

**Fig 1:**
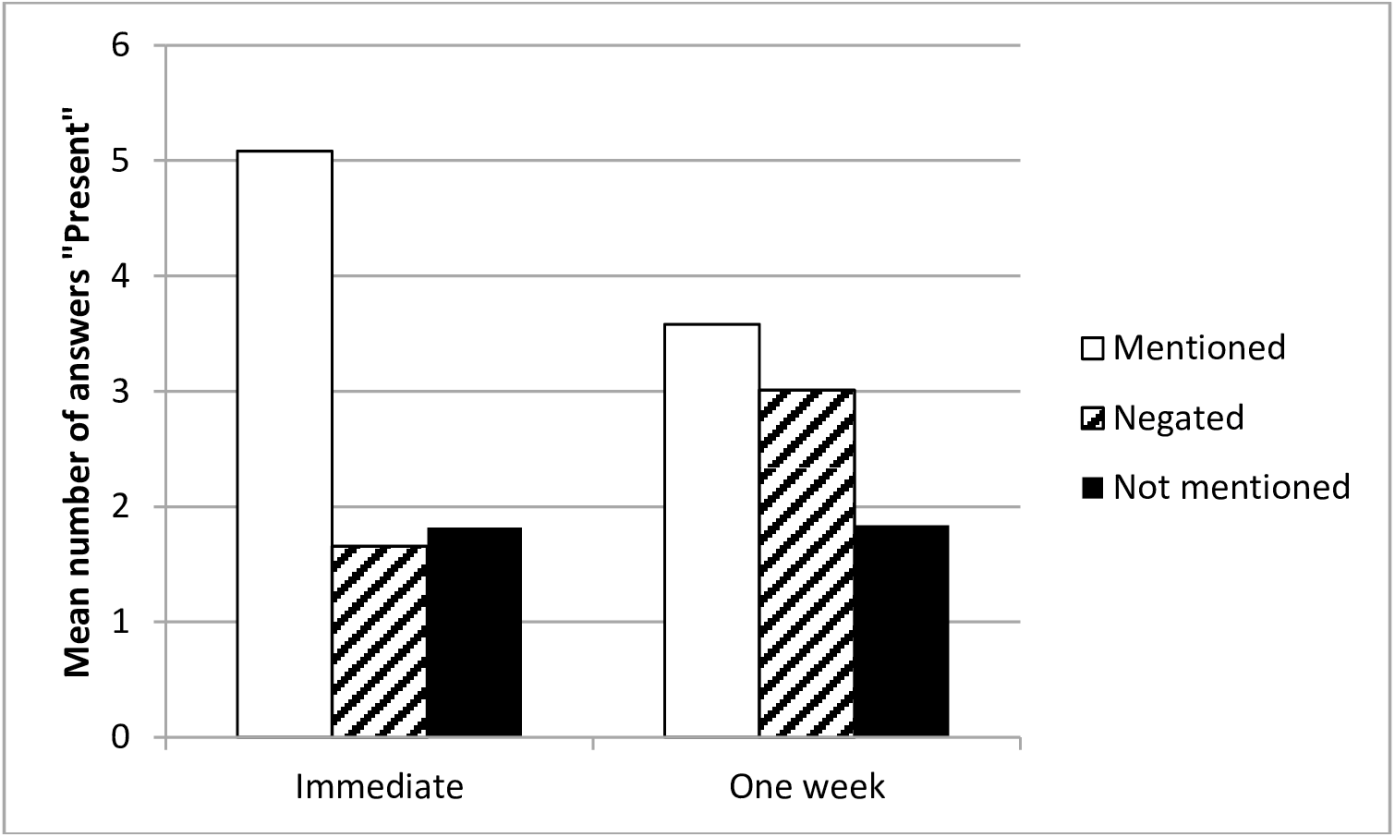
Mean number of ‘Present’ answers as a function of delay and type of information—Experiment 1.

In general, the type of information (mentioned, negated, and not mentioned) significantly influenced the number of ‘Present’ answers, *F*(2, 470) = 211.41, *p* < .001, η^2^_p_ = .47; it was higher in the case of mentioned items than in the remaining two cases. The interaction of this effect with the Delay was statistically significant, *F*(2, 470) = 62.61, *p* < .001, η^2^_p_ = .21 (Fig 1); the analysis of simple effects revealed that in the immediate condition the items mentioned in the description were more often answered as ‘Present’ than were either negated or not mentioned items (respectively: *F*(1, 235) = 507.37, *p* < .001, η^2^_p_ = .68 and *F*(1, 235) = 486.96, *p* < .001, η^2^_p_ = .67). The difference in the number of errors and false alarms (between ‘Present’ answers for the negated and not-mentioned items) was not significant (*F*(1, 235) = 1.25, *p* = .264, η^2^_p_ = .01). In the delayed condition, as compared with the immediate one, the number of correct ‘Present’ answers was smaller (*F*(1, 235) = 117.77, *p* < .001, η^2^_p_ = .33), while the number of errors for negated items increased (*F*(1, 235) = 52.38, *p* < .001, η^2^_p_ = .18). The number of incorrect ‘Present’ answers for the not-mentioned items did not differ significantly between the immediate and the one-week delay conditions (*F*(1, 235) = 0.05, *p* = .822, η^2^_p_ < .01).

The hypothesised NRFM effect was present in the delayed condition; items negated in the description were more often reported as ‘Present’ than were items not mentioned at all, *F*(1, 235) = 31.33, *p* < .001, η^2^_p_ = .12. This result confirms that negated items tend to be remembered as present more often than items that were not mentioned at all. Thus, the hypothesis about the NRFM effect was confirmed. The difference between mentioned and negated items was significant, with more correct ‘Present’ answers than errors, *F*(1, 235) = 6.99, *p* = .008; η^2^_p_ = .03 (although it was not significant in the “Typical” condition; *F*(1, 235) = 1.58, *p* = .210, η^2^_p_ < .01). The difference between mentioned and not-mentioned items was also significant, *F*(1, 235) = 68.93, *p* < .001, η^2^_p_ = .23.

In addition to the significant interaction of delay with type of information, the interaction of type of information with typicality also was significant, *F*(2, 470) = 14.83, *p* < .001, η_p_^2^ = .06. Typical items that were not mentioned were falsely reported as being present more often than were untypical items that were not mentioned, *F*(1, 235) = 34.40; *p* < .001, η^2^ = .13. Thus, the hypothesis expecting more false alarms for typical not mentioned objects than untypical not mentioned was confirmed. The differences between typical and untypical items in the case of mentioned and negated items were not significant (respectively: *F*(1, 235) = 1.55; *p* = .215, η^2^ = .01, and *F*(1, 235) = 0.09, *p* = .765, η^2^ < .01). Finally, the three-way interaction of Type of information, Delay, and Typicality, was not significant, *F*(2,470) = 0.24, *p* = .785, η_p_^2^ < .01. Thus, the hypotheses expecting no difference between typical and untypical items was confirmed.

### Discussion

The participants’ memories were more accurate in the immediate condition; the number of ‘Present’ answers was significantly higher in the case of the actually mentioned items than in the case of negated items or items that were not mentioned. After a week, the number of correct answers dropped, while the number of false alarms for negated items increased (there were no differences between the short and long delays with respect to items that were not mentioned). On the other hand, the data clearly show that the delay did not result in a complete erasure of memory, as the number of correct ‘Present’ answers was higher in the case of items that were mentioned in the source material, relative to the number of errors or false alarms. However, when we take only the typical items into consideration, there was no difference between when the tested items were initially mentioned or negated. This may be due to the typicality effect: in the case of typical items, the false alarms may result from filling memory gaps with ideas consistent with schemata.

The typicality effect for not mentioned items was confirmed; this is yet another replication of this effect. As for mentioned and negated items, it seems that the effects of typicality was counterbalanced by the effects of the script.

The most important result of the present analyses was that there were more false alarms (that is, incorrect ‘Present’ answers for negated items), than there were false positives for items that were not mentioned in the source material. This means that the postulated NRFM effect was present: after a week, negated items (i.e. items that the source material stated were not present) tended to be remembered as having been present to a greater degree than were items that were not mentioned in the source material at all.

In the literature, two models of processing negations are often analysed [22]: the schema-plus-tag model and the fusion model. In recent years the fusion model has been more often discussed as a one-stage model of negation processing [48] or as a dynamic pragmatic model [17] while the schema-plus-tag model is often discussed as a two-step model [49].

The schema-plus-tag model assumes that negation is encoded as a core marked with a negation tag, e.g. the sentence ‘B is not above A’ is represented as a positive proposition accompanied by a negation tag, i.e.: (Not (B above A)). According to the fusion model, the negation operator is ‘fused’ with the core, resulting in an affirmation. For example, the sentence ‘Tom is not guilty’ would be encoded as ‘Tom is innocent’. Experiment 2 of the above mentioned study [22] is particularly relevant to the issue of the effects of negations on memory. The structure of memory errors was clearly different; participants more frequently forgot the negation tag (errors of dissociation) when recalling uni-polar negations compared to bi-polar negations (a bi-polar description has a clear opposite construct, e.g. warm-cold, whereas a uni-polar description does not, e.g. charismatic). The authors interpreted these results within the context of the assumptions of the fusion model and the schema-plus-tag model.

It would seem that the ‘negation related false memory’ effect (NRFM) obtained in our experiment (a significantly greater number of false alarms in the case of negated than not mentioned item) may be explained by the assumptions underlying the schema-plus-tag model. This is related to the fact that negation in Experiment 1 has an exclusionary nature (whereby the negation rules out the presence of something), while it does not indicate any alternative. In other words, it concerns descriptions that are uni-polar, that is, where negation does not result in the assertion of an opposite alternative (e.g. ‘no bread’). In this respect it is similar to uni-polar negation [22]. Errors of dissociation, meaning the separation of the negation marker from the core of the message, are predicted by the schema-plus-tag model and are particularly relevant to uni-polar descriptions. As the authors write [22] ‘According to the schema-plus-tag model, the negation operator could be detached from the core supposition. As a result, individuals may remember the opposite of the intended meaning’ (p. 435).

Thus, it may be assumed that the memory effects of negation presented above are associated with the phenomenon of dissociating the negation tag after the passage of a certain amount of time, which leads to an increased frequency of false alarms, indicating that the negated items were present in the situation mentioned above. However, memory tests after a shorter delay indicated the retention of the negation marker, as expressed in greater participant recall for items encountered earlier and relatively low error rates.

In the next experiment, the NRFM was explored in a sample of children. This is an interesting sample within which to example the NRFM, as detecting the nonexistence of objects is one of the earliest functions of negation during child development [50].

## Experiment 2

The aim of Experiment 2 was to replicate the NRFM effect in an entirely different population, namely, children between 5 and 6 years old, and with different materials. We were unable to locate any past research on the paradoxical effects of negation on memory in children, in contrast to research on children’s understanding of negation, e.g. [19,50,51]. Given the substantially lower cognitive capacity of children compared to adults, the delay between the presentation of the original information and the memory test was reduced from one week to one day. For similar reasons, the typicality factor tested in Experiment 1 also was dropped. Apart from that, the procedures were broadly in line with those of Experiment 1.

### Methods

#### Participants

Forty children were tested; 30 of them were six years old and 10 were five years old. Twenty-five participants were girls and 15 were boys. Most of the children were tested in their kindergarten, except for five who were tested in their homes. Before the study began, parents gave written consent allowing the children to take part in the study. The procedure was approved by the Ethical Committee of the Institute of Psychology, Jagiellonian University in Poland.

#### Materials and procedure

The children were tested individually. They were asked initially if they wanted to take part in an experiment. In order to reduce anxiety and to increase the motivation to participate, the study was presented in a friendly way as a game. As the children were unable to read and write, all the materials, instructions, and questions were presented orally.

After the initial conversation with the researcher, the children listened to a pre-recorded fairy tale (of 220 words, about 2 min) through headphones. The fairy tale potentially presented six critical items (concrete objects) that were typical for the situation described. The six critical items were: carrots, sweets, a bed, a bathtub, a table, and a flowerpot. Two of these items were mentioned in the story, two were negated, and two were not mentioned at all. Full counterbalancing was applied so that each pair of items served equally often as the items that were mentioned, negated and not mentioned.

The memory test consisted of six questions related to all six critical items. The questions simply asked whether a given item was present in the tale, i.e. ‘Was there XXX in Peter Bunny’s house?’ While answering, the children were presented with various pictures illustrating the item. Twenty of the children were given the memory test immediately after listening to the narrative and the other twenty were given the test the next day (The experiment was conducted by Paulina Materna (a student of Institute of Applied Psychology).

#### Results and discussion

The mean number of ‘Present’ answers across the experimental conditions are provided in Table 2.

**Table 2:** Means and standard deviations for ‘Present’ answers across the experimental conditions—Experiment 2.

Delay	Means			Standard deviations			N
	Men	Neg	Con	Men	Neg	Con	
Immediate	1.70	0.70	0.60	0.57	0.66	0.82	20
One day	1.45	1.45	0.30	0.60	0.60	0.57	20
Total	1.58	1.08	0.45	0.59	0.73	0.71	40

Men = mentioned; Neg = negated; Con = control (not mentioned)

**Fig 2:**
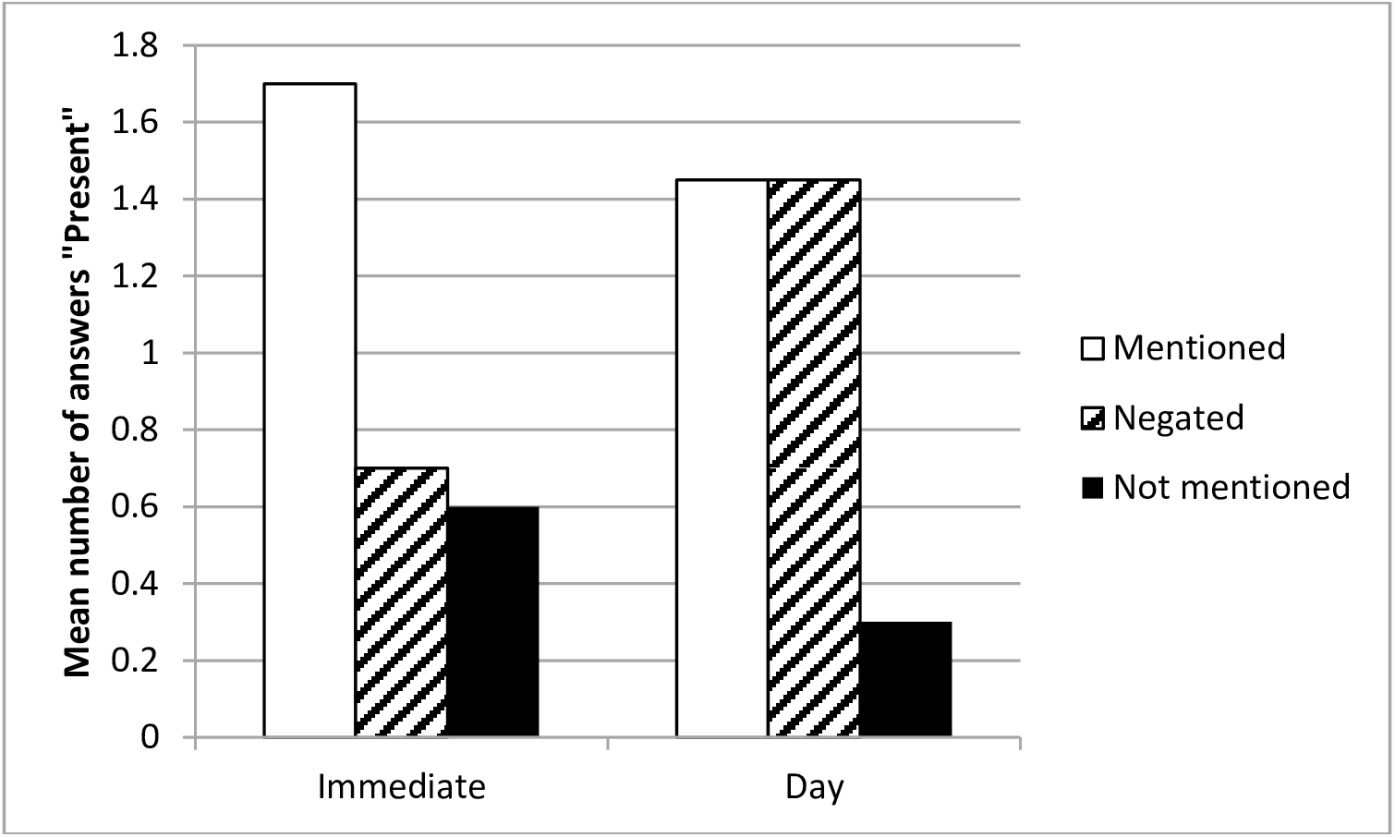
Mean number of ‘Present’ answers as a function of delay and type of information—Experiment 2.

As in Experiment 1, which was conducted with adults, the main effect of negation was significant, with the most ‘Present’ answers being given for items that were mentioned in the narrative, *F*(2,76) = 28.21, *p* < .001, η_p_^2^ = .43. As in the previous experiment, the interaction between type of information and time delay was significant, *F*(2,76) = 7.79, *p* < .001, η_p_^2^ = .17 (see Fig 2). The children were able to give correct ‘Present’ answers in the immediate memory probe condition, and the number of ‘Present’ answers in the ‘Mentioned’ category was much higher than in the ‘Negated’ and ‘Not mentioned’ category, respectively: *F*(1, 38) = 22.35, *p* < .001, η ^2^_p_ = .37; and *F*(1, 38) = 28.42, *p* < .001, η ^2^_p_ = .43. The latter two conditions did not differ significantly from each other, *F*(1, 38) = 0.21, p = .650, η ^2^_p_ = .01. In the delayed condition, however, there were more incorrect ‘Present’ responses for the negated items than for the items that were not mentioned at all (*F*(1, 38) = 27.65, *p* < .001, η^2^_p_ = .42). This means that the NRFM effect was replicated in this sample of children, and the size of the effect was high.

As for the remaining two types of items in the delayed condition, the mentioned and negated items were identified as having been ‘Present’ equally often (*F*(1, 38) < .01, *p* = .999, η ^2^_p_ < .01), and the mentioned items were reported as having been present more often than were non-mentioned items (*F*(1, 38) = 31.08, *p* < .001, η^2^_p_ = 0.45).

In sum, the NRFM effect was replicated with a sample of children. The ‘negation related false memories’ consisting of giving more incorrect ‘Present’ responses when the item had in fact been negated, as compared to when the item had not been mentioned at all. What is more, the difference in the number of errors concerning negated items was not statistically different from the number of correct confirmative answers in the case of items that had actually initially been mentioned. It seems that children are as prone to ‘negation related false memories’ as are adults. This suggests that the NRFM effect may be generalizable across various populations.

As was in Experiment 1, the negation used in Experiment 2 was related to uni-polar descriptions. The question therefore arises whether or not a similar effect would occur when using negation of complementary descriptions (bi-polar descriptions). Complementary negations have a clear state that can be inferred from the negation [22]. Therefore, rather than information about the presence or absence of items, in Experiment 3 we used descriptions of people’s behaviours, which are of a more complementary nature (e.g. if someone did not bring a document from home, this means s/he left the document behind).

## Experiment 3

The aim of Experiment 3 was to replicate the NRFM effect obtained in Experiments 1 and 2. The basic premise of Experiment 3 was the same as that of Experiment 1, but this time, different materials were used. The materials to be remembered in Experiments 1 and 2 pertained to a static description of a house, whereas the materials to be remembered in Experiment 3 were related to human behaviour, namely, driving a car and more specifically, either following or breaking various rules of the road on the part of the driver. Also, instead of differentiating between typical and untypical items, mandatory driving behaviours (prescriptive rules) and prohibited driving behaviours (proscriptive rules) were included.

The first hypothesis was that NRFM, as measured by a greater number of errors for items negated in the source material than false alarm rates for items not mentioned at all, would also occur in the case of human actions. The second hypothesis was that there would be more false alarms (i.e. false positives) for descriptions of behaviours that comply with regulations (mandatory) than for behaviours that do not comply with regulations (prohibited) in the case of the not mentioned items. This was hypothesized because mandatory rules in a way resemble the typicality factor, in that one is typically expected to obey to the rules, not to break them.

### Methods

#### Participants

One hundred and sixty participants, who were mainly first-year psychology students, were tested: 112 women and 44 men (four participants did not indicate their gender). Their mean age was 21.0 (*SD* = 1.9). The participants were volunteers who did not receive compensation for participation, financial or otherwise.

The participants provided verbal informed consent to participate in the study. Written consent was not obtained in order to assure the declared anonymity of the participation. The procedure, including giving verbal consent, was approved by the Ethical Committee of the Institute of Psychology, Jagiellonian University.

#### Materials

1. An audio recording presented a narrative about a man who drives a car and follows or breaks various traffic regulations, e.g. ‘After starting the engine, he did not turn on the headlights but he fastened his seat belt…’ The narrative consisted of about 325 words, and it lasted 3 minutes and 14 seconds. Both mandatory or prohibited behaviour were expressed as affirmations or negations (or not mentioned at all). There were two versions of the text; one included behaviours compliant with the rules of the road, while the other included non-compliant behaviours. As in Experiments 1 and 2, both versions were fully counterbalanced so that each action of the driver would be mentioned, negated, or not mentioned. In sum, there were six versions of the text in both types of behaviours (mandatory and prohibited).

2. A memory test consisted of 15 actions by the driver that were always stated in the affirmative, e.g. ‘he yielded the right of way’, ‘he parked in a restricted area’. Five of the items were actually mentioned in the original narrative, five were negated, and five were not mentioned at all. Depending on the counterbalanced version, a given item might have served for a given participant as a mentioned, negated, or control item (not mentioned). There were two versions of the memory test, one including mandatory items and the other including prohibited items. For each item, the participant had to choose one of three options: ‘Present’ (i.e. the driver performed the action), ‘Absent’ or ‘Not mentioned’. Thus, the final memory test differed somewhat from that used in Experiments 1 and 2, where the participants simply chose between ‘present’ and ‘not present’. However, the dependent variable was essentially the same: it was the number of ‘Present’ answers, which could range from 0 to 15.

#### Procedure

Participants were tested in groups of 4 to 18 persons. The procedure was identical to that used in Experiment 1, with half of the participants taking the memory test after a filler task, and the other half taking the memory test a week later.

#### Experimental design

The design was similar to Experiment 1, with two between-subject factors in the experimental design: delay (immediate vs. one week) and the type of rule in the narrative (mandatory vs. prohibited). Affirmation, negation, and mentioning was manipulated as a within-subjects factor, with each of the participants listening to and responding to the mentioned and negated items, and responding to the not mentioned items. The design was: delay (2: immediate vs. week) × version (2: mandatory vs. prohibited) × type of information (3: mentioned vs. negated vs. not mentioned). Analysis of variance (ANOVA) was used to analyse the data.

#### Results

Table 3 presents the descriptive results for the mean number of ‘Present’ answers across all the experimental conditions.

**Table 3:** Means and standard deviations for the ‘Present’ answers across the experimental conditions–Experiment 3.

		Means			Standard deviations			N
Delay	Version	Men	Neg	Con	Men	Neg	Con	
Immediate	Mandatory rules	3.16	0.81	1.32	1.44	0.91	1.20	37
	Prohibitory rules	3.89	0.78	0.61	1.10	1.14	0.91	40
	Total	3.54	0.79	0.96	1.32	1.03	1.11	77
One Week	Mandatory rules	2.12	2.00	1.42	1.14	1.25	0.98	43
	Prohibitory rules	2.14	1.77	1.33	1.58	1.33	1.17	40
	Total	2.13	1.89	1.38	1.36	1.29	1.07	83
Total	Mandatory rules	2.60	1.45	1.38	1.38	1.25	1.08	80
	Prohibitory rules	3.02	1.28	0.97	1.61	1.33	1.10	80
	Total	2.81	1.36	1.17	1.51	1.29	1.11	160

Men = mentioned; Neg = negated; Con = control (not mentioned)

The main effect of Type of information was significant *F*(2, 312) = 100.53, *p* < .001, η_p_^2^ = .39, as were its interactions: with Delay: *F*(2, 312) = 49.92, *p* < .001, η_p_^2^ = .24; with Version (versions containing mandatory or prohibited actions): *F*(2, 312) = 4.70, *p* = .010, η_p_^2^ = .03; and the three way interaction: *F*(2, 312) = 3.39, *p* = .035, η_p_^2^ = .02. As in Experiments 1 and 2, the results of the immediate memory probe condition were as expected, with correct ‘Present’ answers given much more often for mentioned items than for either negated items, *F*(1, 156) = 196.69, *p* < .001, η^2^_p_ = .56, or for items that were not mentioned, *F*(1, 156) = 168.36 *p* < .001, η^2^_p_ = .52. No NRFM effect was observed in this condition, as the number of false alarms did not differ between the negated and the not mentioned items, *F*(1, 156) = 1.15, *p* = .284, η^2^_p_ = .01.

**Fig 3:**
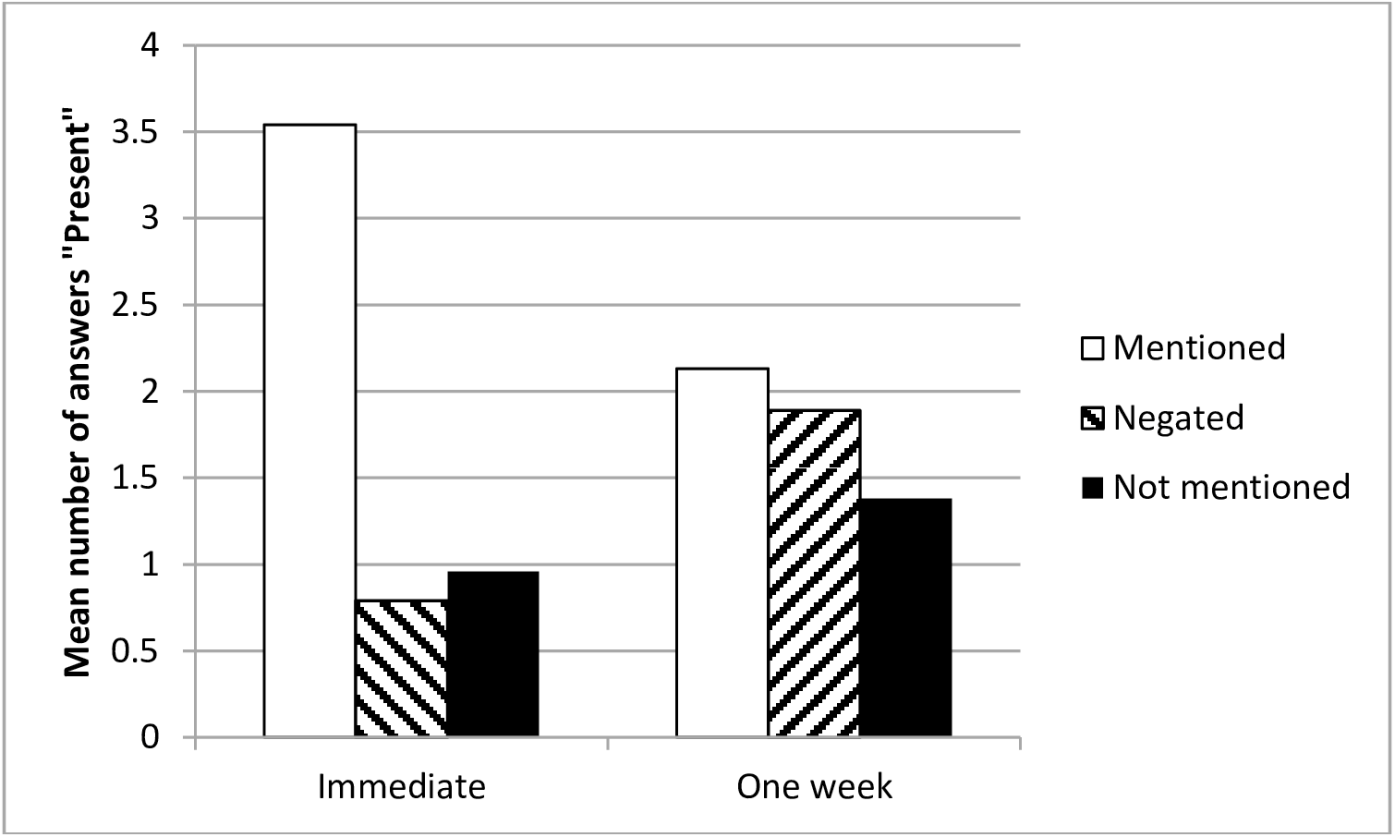
Mean number of ‘Present’ answers as a function of delay and type of information- Experiment 3.

The number of correct ‘Present’ responses fell in the one-week condition, *F*(1, 156) = 44.40, *p* < .001, η^2^_p_ = .22, while the number of errors for negated items rose, *F*(1, 156) = 34.26, *p* < .001, η^2^_p_ = .18, as did the number of false alarms for actions that were not mentioned, *F*(1, 156) = 5.74, *p* = .018, η^2^_p_ = .04. Most important, the number of incorrect ‘Present’ responses in the one-week condition was higher for negated items than for items that were not-mentioned (*F*(1, 156) = 10.72, *p* = .001, η^2^_p_ = .06) (see Fig 3). Moreover, the difference between the items that were mentioned and negated was not significant (*F*(1, 156) = 1.70, *p* = .194, η^2^_p_ = .01). Thus, it seems that negating an item results in just as many ‘Present’ answers as mentioning it does. Finally, there were more correct ‘Present’ responses in the one-week condition for the mentioned items than there were false alarms for the items that were not mentioned *F*(1, 156) = 15.80, *p* < .001, η^2^_p_ = .09.

These effects were moderated by the version of the narrative (mandatory vs. prohibited behaviours), as indicated by the statistically significant three-way interaction (*p* = .035). However, inspection of relevant simple effects revealed that this did not affect the actual NRFM effect, which was present in both versions; furthermore, the mean number of ‘Present’ responses in the one-week condition was not significantly different between the mandatory and prohibited behaviours (the differences for the mentioned, negated, and not mentioned items, respectively, were: *F*(1, 156) = 0.01, *p* = .927, η^2^_p_ < .01; *F*(1, 156) = 0.79, *p* = .384, η^2^_p_ = .01; *F*(1, 156) = 0.14, *p* = .713, η^2^_p_ < .01. The three-way interaction was significant presumably mainly because of the effects related to the immediate memory test, where the number of correct ‘Present’ responses in the case of the mentioned items was higher for prohibited than for mandatory behaviours (*F*(1, 156) = 5.82, *p* = .017, η^2^_p_ = .04), while the reverse was true in the case of the not mentioned items (*F*(1, 156) = 8.46, *p* = .004, η^2^_p_ = .05). There was no significant difference between mandatory and prohibited behaviours in the case of the negated items, (*F*(1, 156) = 0.01, *p* = .909, η^2^_p_ < .01).

Finally, the analyses of the interaction of type of information with the Version revealed that the only significant effect involved the notmentioned items, where behaviours related to breaking mandatory rules were more often falsely reported as present than were behaviours related to prohibitory rules, *F*(1, 156) = 5.53, *p* = .020, η^2^ = .03. The differences between the mandatory and prohibitory rules for the mentioned and negated items were not significant, *F*(1, 156) = 3.24, *p* = .074, η^2^ = .02, and *F*(1, 156) = 0.49, *p* = .485, η^2^ < .01, respectively. Finally, in the long delay, the difference between mentioned and negated items in the prohibitory condition proved nonsignificant (*F*(1, 156) = 1.91, *p* = .169, η^2^_p_ = .01), as did this difference in the case of the mandatory condition (*F*(1, 156) = 0.20, *p* = .656, η^2^_p_ < .01).

## Discussion

As in Experiments 1 and 2, correct ‘Present’ responses were given frequently for mentioned items, but less frequently for negated and unmentioned items. After a one-week delay, the number of correct responses dropped, and the number of both kinds of false alarms rose, obviously due to the fading of memory. In the delayed condition, the number of ‘Present’ responses was higher for mentioned items than for items that were not mentioned. This indicates that despite a relatively complicated narrative (almost twice as long as in the Experiment 1) and a week-long delay, participants were still able to discriminate between what they had heard and what they had not heard. However, this was not the case when items were negated; those items were reported as ‘Present’ with almost equal frequency as those that were actually mentioned as present in the source material. Furthermore, the number of incorrect ‘Present’ responses in the delayed condition was higher for the negated items than for the ones that were not mentioned. This replicates what we have called negation related false memories (NRFM). After some time, when the memory for a given event deteriorates, people may remember negated information as if it had not been negated. In other words, in light of the present results, it seems that mentioning something has the same effect on memory recall as negating it. In this study the negation effect was even more pronounced than it was in Experiment 1, in which the difference between mentioned and negated items was significant only for atypical objects.

There was a difference between mandatory and prohibited behaviours only in the short-delay (immediate test) condition, and it involved the mentioned and not mentioned items. With respect to the mentioned items, there were more correct answers for prohibited behaviours than for mandatory behaviours. This may be because direct information about a driver’s behaviour that is unusual, in that it conflicts with the rules of the road, is remembered more easily. However, with respect to the items that were not mentioned, there were significantly more false alarms for mandatory behaviours than for prohibited ones. This may be explained by the influence of schema, i.e. expectations regarding the driver’s behaviour, namely, that s/he will observe the rules of the road more often than break them.

The results of Experiment 3 on the memory effects of negation are particularly interesting (and perhaps even surprising) considering the assumption about the role of negation, which differed slightly from that in Experiment 1: in Experiment 1 and 2, negation had an exclusionary character; it eliminated the presence of something without indicating any positive content (e.g. information that there was no refrigerator does not indicate that there was anything present instead of a refrigerator). This comes from the uni-polarity of the descriptions in which case the processing of negation occurs following the schema-plus-tag model. In Experiment 3, negation was associated with complementarity. Complementarity is a circumstance in which the negation of one description implies the declaration of another, and vice versa. For example, If ~X, then Y and If Y, then ~X. Around half of the key descriptions were of such a complementary nature (if John did not stop at the red light, that means he drove through the red light; if he did not fail to yield the right of way, that means he yielded the right of way). Thus, the processing of negation in complementary descriptions may be in accordance with the fusion model; that is, negated sentences may be mapped to their affirmative counterparts. Such processing should minimize the occurrence of false memory alarms resulting from the dissociation of the negation marker. It has been found [22] that recalling bi-polar negative descriptions is associated with a smaller number of memory errors consisting of the loss of the negation marker than recalling uni-polar descriptions. However, the memory effects of negation recorded in the one-week delay condition in the present study do not support this claim. These results may indicate that the participants developed a transient representation of the negated items, which could cause false alarms in the delayed condition.

## General discussion

The main aim of this research was to verify whether negating the presence of an object or behaviour has a paradoxical effect whereby participants mistakenly remember that the object in question was actually present or that the behaviour in question actually did occur. This question is not only interesting theoretically, but it also has obvious practical implications, especially (but not only) in the context of applied forensic psychology. When a witness has heard that something did not take place, after some time s/he may falsely ‘remember’ that it actually did take place, with possible serious consequences for the course of justice.

In light of the present experimental findings, we can conclude that such a danger really does exist. We conducted three studies which directly we addressed the question of whether negating an idea can result in subsequently remembering the idea in the affirmative. In all three studies the negated ideas were remembered as having been present more often than when they were not mentioned at all, a phenomenon we called negation related false memories. The effect sizes were considerable according to Cohen’s [46] criteria, but see [52].

Having established this effect, two basic questions arise. First, what are the mechanisms underlying it, or simply put, why does it occur? Second, how can it be prevented, given the possible negative consequences for memory reports?

It is now well established that negation leads to poorer recall, compared to affirmative propositions. There are two main approaches explaining why negation is more difficult to comprehend and remember. The first one concentrates on the greater complexity of the mental processes involved and the second one focuses on the concept of deactivation. Some cases of memory errors certainly can be explained by the simple fact that processing negation is more difficult in general [22,23]. For example, it can be extremely difficult for someone to correctly remember the sentence ‘It is not true that John did not say that he did not finish working on the project that wasn’t his’. Difficulty remembering such a sentence may result simply from the difficulty of understanding its meaning, which is ‘It is true that John said he is still working on somebody else’s project’. However, the results concerning the NRFM effect obtained in the present study cannot be explained this way, as the negative sentences were relatively simple, having one negation at most.

The other explanation assumes that negation may reduce the accessibility of the negated ideas in mental representation [7,26,53,] but see [54]. Psycholinguistic researchers have tended to attribute these elements of negation to the so-called inhibition effect [26], which is also known as the suppression hypothesis [54]. According to these hypotheses, negation (not, no) fulfils the function of an instruction from the sender to the recipient to suppress the negated information [54], i.e. that negation is connected with inhibiting the activation of the negated content. However, the results of our experiments are not consistent with this explanation.

The inhibition effect of negation has been supported in many areas of psychological research on negation, e.g. research on inference processes [55]. The studies most closely linked to the inhibition hypothesis are the experiments in which the negation operator reduced the level of activation of the negated notion and reduced its accessibility [7]. Other studies have demonstrated that when an item is negated, its perceptual representation in the mind is weaker and less obvious, compared to when it is affirmed [56]. The mechanism of inhibition also applies to the effect of negation on memory [26]. However, the notion of inhibition clearly does not explain our results, as participants more often affirmed the presence of items that had been negated, relative to the items that had not been mentioned at all.

It should be stressed that the inhibition effect is by no means a universal explanation of the effects associated with negation, whether in the context of memory research, or outside it. There is research showing that negation does not necessarily suppresses the ideas towards which it is aimed. For example, it has been posited [57,58] that suppression does not take place when the negated concept (e.g. photographs) is represented in the situational model, e.g. ‘Elizabeth burns the letters but not the photographs’. Also, it is not uncommon for negation to be used to activate ideas [59,60]. The retention hypothesis has been presented [54], as an alternative to the suppression hypothesis. According to the retention hypothesis, suppression is not necessary; the sender may intentionally use negation in order to activate the indented ideas. Negation may be used to activate ideas even if the sentence denies the existence of something, e.g. ‘We suffer from shortage in medicines, milk for children, diapers. There are no vegetables, no fruits, no meat and milk products’ (54). This is particularly obvious in the case of ironic statements, e.g. ‘Nothing happened. (…) no officer appeared: not to investigate, not to take testimony’ (p. 236). As Giora points out, this sentence ‘is imbued with negated concepts’ (op. cit. p. 236). Thus, the sender may intentionally increase the availability of ideas by means of using the negation operator.

Other studies, using a priming paradigm, confirm that negated concepts can activate rather than suppress the content to which they are related. Studies of lexical decision priming [54] have shown, among other things, that priming in the form of a negated word (e.g. not sharp) primes a lexical decision (related to the word piercing) similar to a non-negated word (e.g. sharp). Studies using the affective priming paradigm [1] have shown that the priming effect depends on the affective valence of the priming word, regardless of whether the word itself was negated or not. This means that a negation sign preceding a given word does not inhibit its affective valence. A series of studies [61] on the effects of negative injunctions on directing the attention of recipients has shown that negative directives (commands, suggestions, requests) trigger a paradoxical or counter-effective effect; after receiving a request not to pay attention to object X, participants automatically focused their attention on that object (to a similar degree as participants who were asked directly to pay attention to the given object). In some research [62], negating stereotypes enhanced the activation of stereotypes and negative evaluations.

An approach based on the retention hypothesis is much more promising as an explanation of the NRFM effect. This effect is congruent with the hypothesis that negating an idea may make this idea even more distinctive and pronounced. Interestingly, the retention hypothesis has already gained some empirical support in the context of memory, suggesting that information within the scope of negation can be retained in the memory of a recipient. A study showed that correctly responding with ‘no’ to questions about the presence of certain items, which were not presented in a film watched earlier, led–after a time delay–to a greater number of false memory alarms than in the case of absent items about which no questions were asked in the initial recall [26]. According to the authors, this result means that when participants thought about an object and correctly negated its presence in the film, they also created a representation of that object for a moment. That momentary representation was activated during the second recall test and led to a false memory. To some extent, these results are similar to those obtained in the present research–in both, negation led to false alarms more often than when the given idea was not mentioned at all, and the mechanisms by which these results appeared may be similar. The difference is that in the present research the negations were communicated to the participants, whereas in the previous research [26] the negations were generated by the participants themselves.

It is worth mentioning that Experiments 1 and 2 differ from Experiment 3 as regards the function of the negation. In Experiments 1 and 2 the negation had an excluding nature, while it did not indicate any opposite alternative. Therefore, the NRFM observed in these studies may be explained by the schema-plus-tag model, according to which the core supposition could be dissociated from the negation tag after some time. In contrast, in Experiment 3, the negation was associated with complementarity, which means that the negated sentences may be mapped to their affirmative counterparts. However, the NRFM was also present in this case. This means that it may be induced not only by dissociation, but also by activation of mental representations of the negated ideas, in congruence with the theory by Giora et al. [54].

Another explanation of the present results may be based on the two-step simulation hypothesis of negation [49]. According to this hypothesis, when we encounter a sentence denying the existence of some entity, we first construct a mental simulation of this entity and only later turn to simulate the actual state of affairs, that is, the nonexistence of the entity. During the memory task participants may unwittingly access their simulation of the objects or behaviours, initially omitting the negation, as a result of which a false memory of the object or behaviour appears, thereby causing a false alarm.

Finally, it is worth noting that the number of correctly recalled items, in the case of the items that had in fact been initially mentioned, was close to the number of the errors for negated items (the difference was significant in Experiment 1, but not in remaining two experiments). This is obviously due to the decay of memory in the case of the mentioned objects, but it may suggest that in some conditions negating an idea may have comparable effects to mentioning it.

### Limitations and future directions

One of the important questions related to the present research is whether the false memories are indeed induced by the negations. We called the main effect under study ‘negation related false memories’, not ‘negation induced false memories’ because the present effects and procedure do not allow us to conclude that false memories were indeed caused by the negation. It may be that the sole mentioning of an object causes a person to falsely remember it. To resolve this issue, further research is needed with additional experimental conditions including other linguistic operators, especially those that would indirectly suggest nonexistence, e.g. ‘There is a living room in the house and the place would benefit if there were a fireplace as well’.

Due to time constraints, no manipulation check as regards the typicality of the items in Experiment 1 was performed, limiting the conclusions that we can draw regarding this factor. It was, however, of secondary significance for the main aim of the study.

In future research, it may by promising to analyse recognition time, in addition to recognition accuracy. This would allow for the comparison of the time necessary to produce answers in the case of mentioned, non-mentioned and negated items, possibly revealing various reaction times. This would provide further indication of different underlying processes.

The material used in the present research—narratives listing what was or was not in a house, and what a drive did and did not do—are not especially representative of how stories are used in real life. On one hand, it is common in basic research on memory to use artificial ‘laboratory’ materials, such as lists of words or brief stories. The present research was conceived as basic. In future research it will be tested whether context factors, like the perspective of the actor, aims, conflicts moderate remembering negation, correct answers or false alarms.

One of the most important future directions for research on the NRFM effect is research aimed at explaining its underlying mechanisms. The present research demonstrated the existence of the effect, but it cannot determine which of the various theories mentioned throughout the text best explains it. This requires future research. For example, an interesting question is whether or not the NRFM only consists in forgetting the negated particle. This may be analysed by requiring participants to do more sophisticated mental operations in order to answer a question. For example, if the original material contained the sentence ‘John was not texting while driving’, then answering ‘Yes’ to the statement ‘John was texting while driving’ only ‘requires’ forgetting the negating particle. However, if the sentence in the final text would be ‘John wrote a text message while driving’ than committing a false alarm in this case would require some sort of ‘deeper’ information processing than just losing the particle ‘not’.

Also, in the present research, when taking the memory test participants committed primarily one type of error consisting in a false memory alarm (stating that a given element which was in fact negated or not mentioned was present in the original material). We assume that the type of mistakes made can aid in understanding the manner in which negated descriptions are remembered. It may also be fruitful in future research to apply the research paradigm of surface structures—the lowest level of understanding and remembering a text [63]. We assume that the way participants would remember the surface structure would help shed further light on the mechanisms underlying NRFM.

In Experiment 1, the difference between false alarms (recall of negated items) and correct recall (recall of mentioned contents) in the long delay was significant. This means that even in the long term the impact of negation as suppressor of memories worked. This effect was not significant in Experiment 2 (possibly due to power issues) nor in Experiment 3 (both in the prohibitory and mandatory contents). In any case, the NRFM is constituted by the difference between negated and not mentioned items. The fact that, in Experiment 1, there were fewer false alarms in the case of negated items than positive answers in the mentioned ones indicates the possible inhibitory effects of negation. However, there were enough of the subjects to commit more false alarms for negated than for not mentioned items to produce the NRFM.

### Practical implications

The NRFM described in the present study may have significant pragmatic implications: they may help lead to incriminations by innuendo. There is research showing that negations (apart from questions) may serve as innuendos [64,11]. In research by Wegner, participants were shown several newspaper headlines that conveyed different forms of negative information about people. Some headlines were directly incriminating statements (e.g. Bob Talbert Linked with Mafia), others were questions (e.g., ‘Is Bob Talbert Linked with Mafia?’), and others were denials (e.g., ‘Bob Talbert Not Linked with Mafia’). Participants’ impressions of the targets were more negative when they were mentioned in the context of questions or negations, relative to when they were mentioned in neutral headlines. Moreover, both the negations and the questions may have the same effect as directly incriminating statements.

This is bad news for the politician mentioned at the beginning of this article who said: ‘I didn’t beat my wife, I didn’t cheat on her, and I don’t abuse alcohol’. This statement alone can trigger incrimination. After some time, when the public forgets the negations, all that remains is the idea that the politician has indeed committed all of these deeds. In light of the results of the current research, the upset politician would be better advised not to deny anything, but rather to make no comment about the accusations whatsoever. Denying accusations may result in even stronger memory of them on the part of the milieu.

## Supporting information

S1 Table List of words used in Experiment 1. (DOCX)

S2 Table List of actions used in Experiment 3. (DOCX)

S1 Dataset Dataset for Experiment 1. (SAV)

S2 Dataset Dataset for Experiment 2. (SAV)

S3 Dataset Dataset for Experiment 3. (SAV)
