# Supplementary material for: There was not, they did not: May negation cause the negated ideas to be remembered as existing?
Source: PLoS One. 2017 Apr 27;12(4):e0176452. doi: 10.1371/journal.pone.0176452 (PMC5407813; doi:10.1371/journal.pone.0176452)
Supplement: S1 Table — (DOCX) [file pone.0176452.s001.docx]

**S1 Table. List of words used in Experiment 1.**

| Typical | Untypical |
| --- | --- |
| bed | banner |
| bedding | barbed wire |
| chairs | beater |
| curtains | bread oven |
| garage | carousel |
| hall | chapel |
| intercom | clothe dryer |
| lamps | crane |
| living room | ladder |
| mirror | oak |
| refrigerator | plow |
| table | samovar |
| television | sauna |
| toilet | scythe |
| wardrobe | slide |
| washer | tower |
| washing machine | water hose |
| windowsill | well |
