# Supplementary material for: There was not, they did not: May negation cause the negated ideas to be remembered as existing?
Source: PLoS One. 2017 Apr 27;12(4):e0176452. doi: 10.1371/journal.pone.0176452 (PMC5407813; doi:10.1371/journal.pone.0176452)
Supplement: S2 Table — (DOCX) [file pone.0176452.s002.docx]

**S2 Table. List of actions used in Experiment 3.**

| Prohibitory | Mandatory |
| --- | --- |
| beeping the car horn in town | giving the right of way |
| crossing double solid lines | giving the right of way to an ambulance |
| driving against the tide | slowing down at the „dangerous bend” sign |
| driving along a bus line | slowing down at the pedestrian crossing |
| driving back in tunnel | stopping at the „stop sign” |
| overtaking in spite of the “no overtaking” sign | stopping at the level crossing |
| parking at the „no parking” sign | stopping at the red light |
| parking on a place for people with disabilities | stopping at the tram stop |
| passing “no entry” sign | switching to low beam |
| passing an auto giving way to pedestrians | taking the driving license |
| running a read light | taking the vehicle documents |
| stopping on a highway | using the blinker |
| taxting while driving | using the fog lights |
| turning back at the “no turning back” sign | using the low beams |
| using the phone while driving | using the seat belts |
